# Special Issue Editorial: Plant Nitrogen Assimilation and Metabolism

**DOI:** 10.3390/plants10071278

**Published:** 2021-06-23

**Authors:** Fernando de la Torre, Concepción Ávila

**Affiliations:** Departamento de Biología Molecular y Bioquímica, Facultad de Ciencias, Campus de Teatinos s/n, Universidad de Málaga, 29071 Málaga, Spain; fdelatorre@uma.es

**Keywords:** allocation and mobilization, assimilation, biotechnological and agronomical approaches, nitrogen metabolism, nitrogen use efficiency

## Abstract

Nitrogen is an important macronutrient for plant growth and development. Research has long been carried out to elucidate the mechanisms involved in nitrogen uptake, assimilation, and utilization in plants. However, despite recent advances, many of these mechanisms still are not fully understood. In this special issue, several research articles and two reviews, all of them aiming to elucidate some specific aspects of nitrogen (N) metabolism, are presented. Together, the articles in this issue provide a state-of-the-art perspective on important questions related to nitrogen metabolism in photosynthetic organisms, highlighting the fundamental importance of research in this field.

The living organisms present in nature have an enourmous versatility to metabolize most nitrogenous compounds. The paper by Bellido-Pedraza et al. (2020) [1] reviews the N cycle in the model alga *Chlamydomonas reinhardii* [2,3] and its participation in different reactions of the N cycle [4]. The authors made particular emphasis of their description of new putative genes encoding enzymes involved in N-transforming reactions as NO and N_2_O production and their occurrence in other algae genomes. Very interestingly, this review also illustrates the mutualistic interactions between algae and bacteria during the ammonification process.

The existing interconnections between flavonoid and isoflavonoid biosynthetic pathways with N metabolism were updated by García–Calderón et al. (2020) [5]. The authors placea special emphasis onresults obtained using photorespiratory mutants deficient in the key enzyme glutamine synthetase [6]. The paper provides evidence showing that an enhancement of isoflavonoid, compared to standard flavonol metabolism, occurs in *Lotus* plants under abiotic stress conditions. The use of co-expression networks has allowed the authors to progress in the study of the regulation of the process [7].

The selected research articles in the issue provide an update of progress made in different aspects of N metabolism, such as N assimilation in adverse conditions, the implication of C in N assimilation, and studies on the use of different N sources.

## 1. N Assimilation in Adverse Conditions

The study of new varieties of crops with improved characteristics under conditions that limit plant productivity is one of the topics that have always concerned researchers in plant N metabolism, and this has been addressed within this special issue in two research papers. Environmental conditions, such as limitations of water seriously affect N assimilation and the accumulation of plant biomass. This important topic for N homeostasis, considering current climate changing conditions, is addressed in the article by Xia et al. (2020) [8] using as a model system a forage crop with strong resistance and wide adaptability to drought stress, sweet potato. Reduction of plant biomass under drought stress together with the down-regulation of N uptake genes is a general behavior in most crops [9]. This work concludes that NR is critical for drought response in leaves, while in roots GS and GOGAT they are the fundamental determinants. The authors claim that this could be a main factor to ameliorate the growth of sweet potato under drought stress.

Using a high-salt and boron-tolerant maize ecotype, Fuertes-Mendizabal et al. (2020) [10] evaluate the effect of boron at high and low salinity conditions on N metabolism. Under low salt conditions, the presence of excessive boron stimulates higher nitrate and ammonium mobilization to leaves, increasing nitrate reductase (NR) activity but not glutamine synthetase (GS). The authors describe that the enhancement of NUE by boron application would contribute partially to maintain the biomass production in this ecotype. The increase of major amino acids detected, such as alanine and serine, would indicate a photoprotective role of photorespiration under low-salinity conditions. 

## 2. Carbon Supply and Its Implication in N Assimilation

A better understanding about how C sources can modulate N uptake is useful for improving NUE [11]. Two articles in this special issue address this topic.

González-Hernández et al. (2020) [12] have studied the effect of sucrose, glucose and 2-oxoglutarate on nitrate nutrition. Their results contribute to a better understanding of how these C sources can modulate N uptake and the expression of C/N genes, auxins, and antioxidants. In the light of the results obtained, the authors propose a potential impact for improving NUE in tomato plants.

In the paper by Cañas et al. (2020) [13] it is shown how the overexpression of NADH-GOGAT in maize plants is detrimental for shoot biomass production but does not affect kernel yield. A decrease in kernel production was observed when NADH-GDH was pyramided to NADH-GOGAT and NAD-IDH. This decrease could not be restored even when additional cytosolic GS activity was present in the plants overexpressing the three enzymes producing 2-oxoglutarate. The study suggests that 2-oxoglutarate synthesis is a key metabolic step acting at the interface of carbohydrate and amino acid metabolism. The accumulation of 2-oxoglutarate induces an imbalance of primary carbon and N metabolism that may be harmful for maize growth.

## 3. N Availability: The Use of Different N Sources

The availability of N, and how it affects plant development, has been described in different species.

Understanding N metabolism for improving the mineral nutrition of conifers is the aim of the paper by Ortigosa et al. (2020) [14]. They studied the metabolic changes in maritime pine seedlings using two alternative sources of inorganic N: nitrate and ammonium. The N status was characterized through biomass analyses using different biochemical and molecular markers together with ^1^H-NMR metabolic profiles. The differences in the allocation of nitrate and ammonium serve the authors to suggest an additional function of these N molecules as metabolic buffers to prevent interference with photosynthetic metabolism in pine seedlings.

The variation of N metabolism has been addressed by Iqbal et al. (2020) [15] in cotton, a highly sensitive plant species to N supply. This study explores the intraspecific variation among four cotton genotypes in response to various N supplies, in order to identify the most distinct N-efficient genotypes and their NUE-related traits in hydroponic culture. This work also describes how N uptake and NUE enhancement may be directly related to the mobilization of N from the shoot to roots due to the observed high shoot GS activity.

The effects of N deficiency in radish sprouts is approached at the metabolomic level by Baek et al. (2019) [16]. The authors characterize a total of 81 metabolites including organic and inorganic acids, amino acids, sugars, sugar alcohols, amines, amide, sugar phosphates, policosanols, tocopherols, phytosterols, carotenoids, chlorophylls, and glucosinolates. Their results indicate that, in radish sprouts, the response to N deficiency occurs quickly and dynamically by increasing the content of soluble sugars and γ-tocopherol, which acts as a defense mechanism after the germination of radish seeds.

Symbiotic interactions between plant and root-associated N fixing bacteria enhance plant growth [17]. The article by Calubi Pereira et al. (2020) [18] studied the response of two varieties of corn to the symbiotic interaction with the N-fixing *Azospirillum*. Most positive outcomes obtained in the area have been partly attributed to the genotypic variation of the species used. Using a metabolomic approach, the authors examined the composition the root exudates in relation to the genotypic background of two corn hybrids. The results corroborate that the root exudate composition is affected by the genotypic background and likely reflect the specificity of the transcriptional signaling involved in the mutual recognition between *Azospirillum* and its host plant.

When an excess of N is available, plants can assimilate and store it directly as free arginine, the amino acid with the highest N content and a major component of storage proteins in the bark and the seeds [19,20]. Arginine is also a precursor for the biosynthesis of polyamines, nitric oxide, and a wide range of nitrogenous compounds [21]. However, the enzymes involved in the arginine metabolic pathway have been poorly characterized at the biochemical level in plants. Urbano-Gámez et al. (2020) [22] have recombinantly produced these enzymes and determined their molecular and kinetics properties. The results provide new insights into the regulation of the pathway and suggest that ornithine transcarbamylase is a regulatory step of the ornithine pools in the plastid for arginine biosynthesis and in the cytosol for the synthesis of other nitrogenous compounds.

As summarized above, the collection of 11 articles that make up this special issue devoted to N assimilation and metabolism effectively demonstrate a range of contemporary approaches to investigate key physiological processes involved in development, metabolism, and responses to abiotic factors related to N. Overall, these papers underscore the progress that has been made towards a better understanding of different processes related to the assimilation and homeostasis of this important macronutrient. In the coming years, additional studies are expected covering a broad range of approaches and species related to N metabolism in particular and its impact on plant productivity.
